# Hospital Accounts and Finance

**Published:** 1922-12

**Authors:** Joseph E. Stone

**Affiliations:** (Accountant, St. Thomas's Hospital, London, S.E.)


### HOSPITAL ACCOUNTS AND FINANCE.

### REAL ECONOMY AND HOW TO SECURE IT.

By **JOSEPH E. STONE, F.S.A.A., F.S.S., F.R.Econ.S.** (Accountant, St. Thomas's Hospital, London, S.E.).

NOT more than 25 years ago the typical hospital organisation was one with a relatively small amount of invested capital and annual income and expenditure. Its problems of management, judged by present standards, were not especially complex, and the management committee usually found little difficulty in keeping themselves familiar with most of the financial affairs of the hospital they managed and exercising some kind of control over expenditure. It was rarely that the scope of the activities of any single hospital was so wide as to vitally affect any large community interest. From this it follows naturally that the accounts required to record its financial transactions were of a fairly simple type, and were of interest only to a very few persons. The conventional financial report setting out the cash received and disbursed was found to be entirely adequate to the needs of the typical hospital enterprise of the period. Hospitals have, however, moved with the times, and the growth in size and of the activities of the modern hospital has resulted in a greater complexity of business organisation, necessitating the introduction of a more up-to-date and scientific system of accounting, in order that information of real value to the management committee may be available to assist them to efficiently carry out their duties, and also for the purpose of keeping the public constantly informed of the cost of progress to the hospital. A complete system is necessary, and such a system of accounting is justified in institutions such as hospitals, where public money is entrusted to others for disbursement. The governing authorities of hospitals are, in effect, trustees to the public for the funds they have contributed, and, acting in that capacity, they have two important duties to perform:—

1. To account strictly for all monies entrusted to their charge.

2. *To secure that in spending such monies the greatest economy consistent with efficiency is exercised.*

Economy is a word which appears frequently in reports of hospital meetings, conferences, and in the published annual reports, but in constant reiteration it may lose its significance. Too often it is ill used, as when it is intended to signify only reductions in expenditure instead of retaining its rightful inheritance, which comprises, *inter alia,* full value for money spent. This is the point it is desired to emphasise in this article. *Reduction of expenditure and economy are not one and the same thing.* It is harmful to throw money away just because one has it, or to cut expenses in an emotional reversal of the same spirit; both are actions of unreason, and perhaps the second is the more harmful, because it prevents any progress being made at all. Therefore in approaching the question, "How can we reduce expenditure?" it is necessary to adopt a sane attitude. This will follow automatically if, before we begin the cutting process, we pause long enough to agree that, after all, we are not concerned with expenses as such nor with saving money as such, but with *gaining the proper relation between expenses and services obtained by such expenses*. This is the basis of all pruning of expenditure. Expenditure is high if it does not contribute in adequate proportion to the eventuality of a fair return. Therefore, the problem of efficient and economical hospital administration is to cut out all expenditure which does not make an adequate contribution in result. If an expense does not serve, it is high, however small the amount; if it does serve, it is high only if the service rendered is less than the expenditure ought to have realised. When we start to cut expenditure we cannot say, "Let's reduce this or that item so much per cent."; we cannot by just looking at a mere money figure be dogmatic and say, "This is too high; we must cut it down." To set about cutting expenses in any such manner is like throwing the best china out of the window and tenderly conveying the feather bed downstairs.

Some costs are high, but they may be essential for carrying on the work; we must, therefore, give due consideration to the fact that every cost is purely relative, and from this it follows that no expense should be considered out of its setting. We have to find out where the increases take place, and in this connection knowledge of the bulk expenditure on any section of the expenditure headings is useless; we must get down to the service or unit. In the first place, we shall require a detailed statement of all expenses, for it may be that they have not been uniform; perhaps one or two grouping items in a series are alone accountable for increases. We may find from these details that our materials are much higher than they ought to be; that we have bought unwisely ahead at high prices. Instances such as these show clearly that the answer to the question we have set ourselves involves much more than getting some expense reports and then using a blue pencil more or less haphazard. This is the wrong way to reduce expenditure; such a method is likely to cause friction, retard progress and make for less efficient administration. *The proper thing to do is to lower expenses in cost per unit.* Here we have a definite basis to work upon. We cannot intelligently raise or lower wages without an exact knowledge of costs. It is not enough merely to know that costs are high, but it is absolutely necessary to have them in such arrangement that constant analysis and comparison may be made, so that increases are located almost immediately and the necessary investigation commenced to ascertain the cause. Conversely, when reductions in expenditure are rendered necessary, we shall know how and where to set about making them, because reliable data will be to hand. In this connection a well-planned costing system in conjunction with statistics relating to consumption—both are of equal importance—is the surest of all preventives of disproportionate rises in costs and over-expenditure or extravagance. In fact, an accurate knowledge of costs, &c., is fundamental in the discovery of extravagance. Further, it is the preliminary to the most important and at the same time the most neglected feature of hospital accounting and administration—the Budget. (An article on this subject appeared in the November issue of this journal.—Ed.)

A step towards the inauguration of a costing system is that of departmentalisation of expenditure—one of the essentials of efficient control of expenditure. Under this method all expenditure, no matter from which department the order was sent or the money paid, would be brought into the accounts and charged in detail against the ultimate, and not only the immediate object. For instance, money spent on wages would be charged first to wages account, and then transferred through a wages analysis journal to the departments, &c., responsible for having incurred the expenditure under the heading "Wages." All items now shown in the income and expenditure account would be treated in the same manner, so as to present an amplification and detailed explanation of all such items.

With the knowledge of the working costs of each and every department the management committee are provided with an instrument of control second only to that of the Budget.

*The accounts would then be of real value.* They would show the cost of the unit of service, the total cost of the service, and the final accounts would show the results as a concrete whole. It would then be possible to study not only the all-in cost of treatment, but the cost of each separate stage in the treatment. When in thorough working order and supported by the management committee such a system would provide great opportunities for the practice of economies. It would provide something more than a mere summary of totals; its functions would become vital, and its sphere of usefulness immeasurably increased.

In conclusion, it is as well to remember that the object of accounting in hospitals is not merely the keeping of certain books and the entering therein of a number of transactions under specified headings, but rather is it to express clearly by comparative analysis the actual effect of the financial transactions and to aim at presenting the final accounts in such a way as will enable the financial results to be readily perceived and understood by the lay mind, and to furnish reliable data that will act as a guide in connection with transactions of a similar kind in future. Our first consideration is, and must always be, efficiency; but there is no reason why, if the proper relation is secured between expenses and services, this efficiency cannot be combined with economy.

---

When the enlargement made possible by the Red Cross grant of £10,000 shall have been completed, Lincoln County Hospital will possess 150 beds. Unemployment has much affected local support, and postponed the starting of a contribution scheme.

